# Closing the (incarceration) gap: assessing the socio-economic and clinical indicators of indigenous males by lifetime incarceration status

**DOI:** 10.1186/s12889-020-08794-3

**Published:** 2020-05-18

**Authors:** Stephane M. Shepherd, Ben Spivak, Linda J. Ashford, Isabel Williams, Justin Trounson, Yin Paradies

**Affiliations:** 1 Centre for Forensic Behavioural Science, Swinburne University of Technology, 1/582 Heidelberg Rd, Alphington, Victoria Australia; 2 Department of Psychology, University of Warwick, University Rd, Coventry, UK; 3 Alfred Deakin Research Institute for Citizenship and Globalisation, Deakin University, Burwood, Victoria Australia

**Keywords:** Indigenous Australians, Closing the gap, Risk factors for offending, Incarceration, Aboriginal

## Abstract

**Background:**

Approximately 1 in 5 to 1 in 6 Indigenous Australian males are currently imprisoned or have previously been imprisoned. Recent work has also pointed to a widening socio-economic gap within the Indigenous population. Given the myriad social, wellbeing and environmental risk factors associated with justice-involvement, it is conceivable that incarceration may contribute to the increasing disparities found within the Indigenous population. This study aimed to explore the presence and extent of an ‘incarceration gap’ within the Indigenous population and to uncover which social factors characterise the disparity.

**Methods:**

The study utilised data from the 2014–5 National Aboriginal and Torres Strait Islander Social Survey (NATSISS). A number of socio-economic, environmental and clinical factors were compared by life-time incarceration status. Chi-square tests were used to examine the association between incarceration status and each of the comparison variables.

**Results:**

Disparities were observed within the Indigenous Australian population across a number of important health and socio-economic markers by incarceration status - the most pronounced being for educational obtainment – year 10 completion (Never incarcerated 73%, Ever incarcerated 50%), labour force participation (Never incarcerated 56%, Ever incarcerated 26%) and drug/alcohol problems (Never incarcerated 7%, Ever incarcerated 29%). Never-incarcerated Indigenous males yielded aggregate proportions across numerous variables that approximated or matched general Australian population estimates.

**Conclusions:**

There appears to be evidence for a substantial ‘incarceration gap’ within the Indigenous Australian population.

## Background

In 2008, the Council of Australian Governments launched “Closing the Gap” (CTG), a nationwide strategy to address the inequality in health and education outcomes between Indigenous and non-Indigenous Australians [1]. A number of targets were delineated to address Indigenous disadvantage and reduce disparities in early childhood education, school attendance, literacy and numeracy attainments, employment outcomes and mortality rates. A decade on, progress has been ostensibly negligible, though some gains have been realised in two target areas (early childhood education, year 12 attainment) [2]. There are notable challenges to gauging progress including obtaining accurate data and the changing demographics of both Indigenous and non-Indigenous populations [3, 4]. Moreover, the targets are dynamic in nature – specific non-Indigenous population markers are simultaneously improving, hindering parity [2]. The CTG framework has also been critiqued on a number of fronts including its assimilationist nature, in which Indigenous people are measured by their similarity to non-Indigenous Australians as the benchmark, an outwardly top-down design [5, 6], an incapacity to contend with structural racism [6], and an over-emphasis on the gap between Indigenous and non-Indigenous populations (‘between gap’) in preference to a broadening gap between disadvantaged and non-disadvantaged Indigenous Australians (‘within gap’). Notably, proposals to include justice targets in the CTG framework have received growing support and political momentum [7, 8]. A number of draft justice targets were proposed in 2018 aimed at reducing the number of Indigenous Australians in the criminal justice system [2, 9]. Rates of Indigenous incarceration have in fact increased since CTG was established more than 10 years ago [10]. And despite an overall drop in the numbers of young people under criminal justice supervision over the past 5 years, Indigenous rates compared to non-Indigenous rates have increased [11].

Explanations for Indigenous over-incarceration are well documented. Proximal factors include substance abuse, low educational obtainment, child maltreatment, exposure to violence, unemployment, family/neighbourhood dysfunction, anti-social behaviours, negative peer group influence, mental disorder and financial strain [7, 12–15]. Representative surveys of Indigenous prisoners underscore the commonality of these factors [16–20]. Justice system contact is also associated with poorer health outcomes including a higher risk of mortality [21]. It has been argued that many of the above factors are underpinned by historical injustices and intergenerational marginalisation, consigning many Indigenous people to underprivileged circumstances and an oppressive relationship with a justice system characterised by institutional racism [22, 23]. Socio-economic challenges perhaps explain to a large extent, contemporary Indigenous over-incarceration - the prison population at large, disproportionately comprises individuals from disadvantaged environments [16]. Yet despite these enduring social challenges, emerging trends underscore the improving economic position of Indigenous Australians. Although still below non-Indigenous levels, Indigenous incomes at the aggregate level are increasing faster than for the non-Indigenous population [24]. Moreover, Indigenous poverty in urban areas has dropped substantially to the point where income parity between Indigenous and non-Indigenous populations in major cities is expected before the year 2040 [24]. The collective economic progress, however, is not befalling all localities. The poverty rates for Indigenous people in remote areas have increased despite income improvements at the aggregate level and significant income increases among the top 10% of the Indigenous income distribution [24]. It has now been noted that income inequality is more pronounced within the Indigenous population than within the non-Indigenous population [24]. Furthermore, the life expectancy of Indigenous Australians decreases by remoteness [25]. This poses an additional challenge for CTG targets as it demonstrates that like many other growing populations, the Indigenous population is complex, dynamic and markedly heterogeneous.

Another potential divergence within the Indigenous population is the ‘incarceration gap’. As of December 2018, there were 11,792 Indigenous Australians in custody [26]. This total considerably increases if we were to include members of the Indigenous population who have previously been incarcerated. For example, almost 15% of the male Indigenous population report ever being incarcerated [27] and 3% are currently incarcerated [26]. This means that roughly 1 in 5 to 1 in 6 Indigenous Australian males are currently imprisoned or have previously been imprisoned. Given the myriad social, wellbeing and environmental risk factors associated with justice-involvement, it is conceivable that an ‘incarceration gap’ may explain to some degree the increasing disparities *within* the Indigenous population. As such, it is of interest to ascertain the presence and extent of an ‘incarceration gap’ and to uncover which social factors characterise the disparity.

This study utilised data from the 2014–5 National Aboriginal and Torres Strait Islander Social Survey (NATSISS). The NATSISS is a representative survey of individuals of Indigenous origin that collects information on a range of social, cultural, environmental and economic markers [27]. Several forensic-related analyses have previously been conducted on various iterations of the NATSISS, which is compiled every 6 years. These studies have explored factors associated with offending [28–31] and violent victimisation [32, 33]. The variables chosen in the current study reflect demographic information and domains (i.e., education/employment, mental health/disability, substance use and exposure to violence) that have demonstrated associations with offending in prior literature [14, 15, 34]. Other variables of interest (i.e., participation in cultural activities, trust in institutions, experiences of racial discrimination and community agency) were included based on their unique relevance to Indigenous Australians [35–38].

We expect to find evidence for an ‘incarceration gap’. Pronounced differences between Indigenous NATSISS respondents by incarcerated status are expected across a number of proximal risk factor domains including educational obtainment, employment, substance use, mental health concerns, services trust/access and exposure to violence. We expect to find fewer differences across distal factors such as cultural participation and racial discrimination.

## Methods

### Materials

A number of variables were extracted from the NATSISS in order to identify differences across socio-economic, and health and wellbeing indices by reported lifetime incarceration status. The following variables utilised in the analysis are listed below. Variables were either dichotomous or in Likert scale form. Data was restricted to male respondents as the small number of ever-incarcerated females would likely render the data unreliable when employing the Australian Bureau of Statistics Table Builder.

#### Demographic variables

Marital Status, Aboriginal and/or Torres Strait Islanders only in household, Section of State (i.e., major urban, rural etc.).

#### Socio-economic variables

Educational obtainment (Completed year 10), Labour force status.

#### Health variables

Personally experienced serious illness/disability in past 12 months, Whether has been diagnosed with a mental health condition, alcohol/drug related problems in last 12 months, and whether seen doctor in last 12 months for own health.

#### Psychological distress

The NATSISS psychological distress variable was measured using the modified Kessler-5 (K5), a subset of five questions from the Kessler Psychological Distress Scale-10 (K10). The modified K5 was adapted for use in Aboriginal and Torres Strait Islander population surveys [39] It has been administered to Aboriginal and Torres Strait Islander cohorts in both general [40] and custodial [41] populations. Questions canvass whether participants had felt nervous, without hope, restless, sad or if ‘everything was an effort’, over the past 4 weeks. All items were scored on a five-point scale (1 = none of the time, 2 = a little of the time, 3 = some of the time, 4 = most of the time, and 5 = all of the time). Scores between 12 and 25 are indicative of high/very high levels of distress. Score between 5 and 11 are indicative of low/moderate levels of distress and scores below 5 indicate no significant stress.

#### Social participation

Whether participated in selected cultural activities in last 12 months, how often feels able to have a say within community on important issues, and how often feels able to have a say with family and friends on important issues.

#### Social challenges

Whether ever removed from natural family, whether unfair treatment in last 12 months because Aboriginal/Torres Strait Islander, whether avoided situations due to past unfair treatment because Aboriginal/Torres Strait Islander, whether experienced physical violence in last 12 months, and whether has problems accessing services.

#### Trust in Institutions

Level of trust in police in local area, level of trust in police outside local area, level of trust in own doctor, and level of trust in hospitals.

### Procedure

Statistics were compiled using the 2014/15 National Aboriginal and Torres Strait Islander Social Survey (NATSISS). The 2014/15 NATSISS was a national survey of individuals of Aboriginal and Torres Strait Islander origin that collected self-reported information on demographic, social, environmental and economic characteristics [27]. It was developed in consultation with representatives from government agencies, peak Aboriginal and Torres Strait Islander groups, and prominent Aboriginal and Torres Strait Islander academics and research bodies [27]. The survey scope includes all Aboriginal and Torres Strait Islander people who were residents of private dwellings in Australia [27]. Data was extracted using Census Table Builder, which allows for registered users to construct tables of census data. Census Table Builder presents weighted NATSISS data in order to create population estimates. The overall sample size of the NATSISS was 11,178 - this is naturally smaller than the overall Australian Indigenous population which is estimated to be almost 800,000 [42]. The NATISISS survey is estimated to under-represent 6% of the Indigenous population. To account for exclusions, the final sample is weighted. To provide population estimates, each NATSISS response is first weighted by the inverse probability of being selected for the survey. For example, if a person had a probability of 1 in 20 of being selected for the survey, their response was weighted to represent 20 individuals. Probability estimates were based on the ABS Aboriginal and Torres Strait Islander population Estimates and Projections 2006–2031, a set of population estimates using the 2016 Australian Census [27]. Weights were calibrated by state, remoteness, sex, age group and Torres Strait Islander status. To avoid identification of individual responses, data is perturbed by slightly adjusting count values through the introduction of small random errors. The perturbation of data does not distort the overall pattern of counts produced in a table, except in cases where cells contain a very small number of observations (e.g. *n* < 3).

### Statistical approach

The Australian Bureau of Statistics (ABS) TableBuilder platform permits users to extract cross-tabulations of simple aggregate information. As such, chi-square tests were used to examine the association between incarceration status and each of the comparison variables examined. Given the large sample size, statistical significance was assured for most comparisons. As such, Cramer’s V was employed to assess the size of associations. Cramer’s V can be interpreted as the percentage of maximum possible variation between two variables and varies from 0 to 1 (with 0 indicating no association and 1 indicating complete association). All analyses were conducted at the national level.

## Results

The sample size represents approximately 336,000 Indigenous males after taking into account both the weighting of results to generalise to the total population, and the random adjustment of cells to prevent the identification of individuals. The number and percentage of Aboriginal respondents was 305,200 (90.5%); for Torres Strait Islander respondents the number and percentage was 17,700 (5.3%); and for respondents reporting both ancestries, the number and percentage was 14,200 (4.2%). The age range included respondents 15 years and over.

**Fig. 1:**
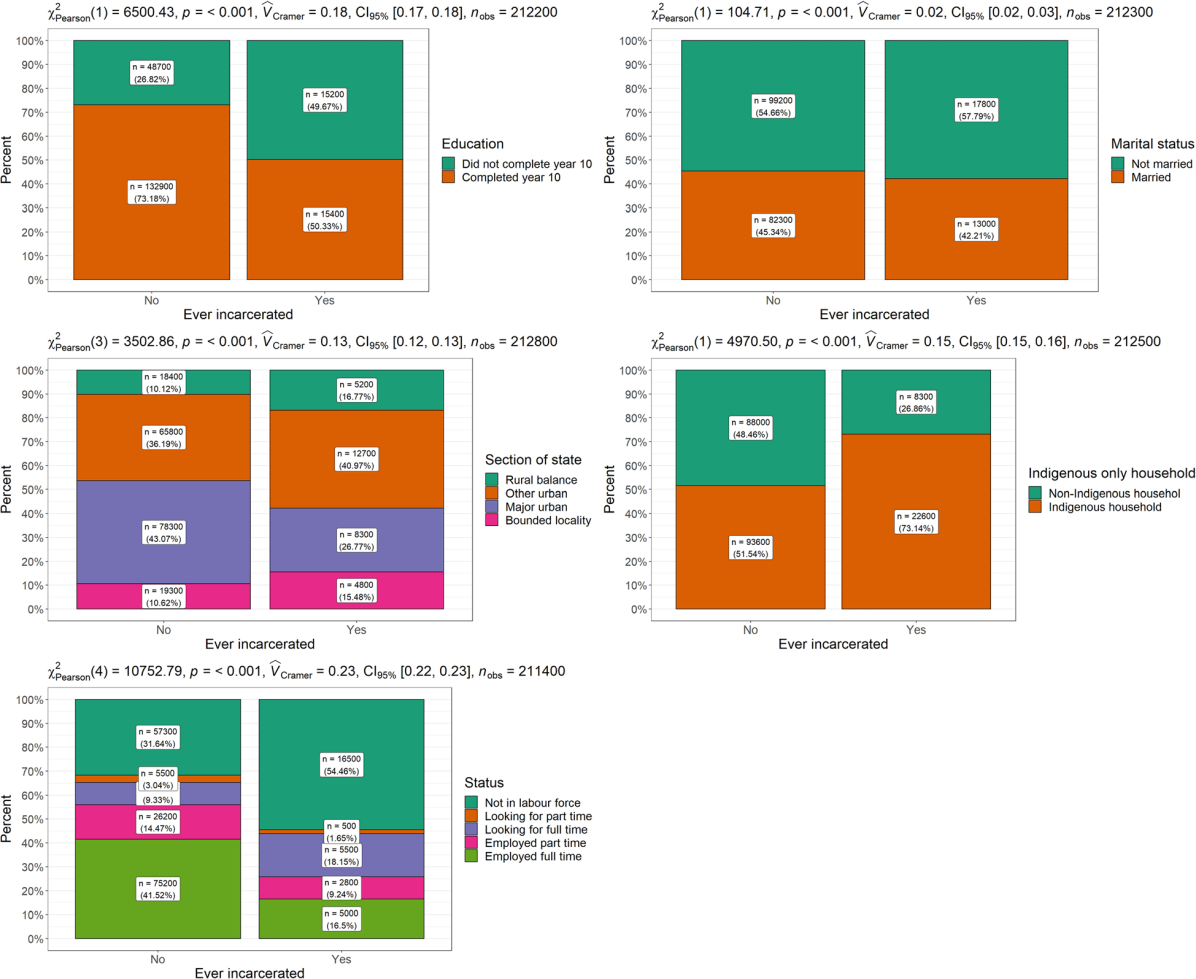
Demographic and socio-economic variables by incarceration status

Table 1 and Fig. 1 present demographic and socio-economic comparisons by incarceration status. All factors examined were significantly associated with incarceration. Nearly three quarters of individuals who reported no experience of incarceration reported completing year 10. In contrast, just over 50% of individuals reporting an experience of incarceration, reported completing year 10. The never-incarcerated group also reported much higher levels of full-time employment (41.5%) compared to just 16% of ever-incarcerated respondents. Marital status was comparable across groups, with considerably fewer mixed Indigenous/non-Indigenous households among the ever-incarcerated group. More never-incarcerated individuals resided in major urban areas whilst ever-incarcerated individuals had greater representation in remote/smaller jurisdictions.

**Table 1:** Demographic and socio-economic variables by incarceration status

	Ever-incarcerated *N* (%)	Never-incarcerated *N* (%)	*Χ*^2^ (*p* value)	*V*
Education				
Did not complete year 10	15,200 (49.7)	48,700 (26.8)		
Completed year 10	15,400 (50.3)	132,900 (73.2)	6447.7 (<.001)	.18
Marital status				
Not married	17,800 (57.8)	99,200 (54.7)		
Married	13,000 (42.2)	82,300 (45.3)	223.7 (<.001)	.02
Section of state				
Major urban	8300 (26.8)	78,300 (43.1)		
Other urban	12,700 (41.0)	65,800 (36.2)		
Bounded locality	4800 (15.5)	19,300 (10.6)		
Rural balance	5200 (16.8)	18,400 (10.1)	3502.9 (<.001)	.13
Indigenous only household				
Non-indigenous individuals in household	8300 (26.9)	88,000 (48.5)		
Only indigenous individuals in household	22,600 (73.1)	93,600 (51.5)	4970.5 (<.001)	.15
Labour force status				
Full time employed	5000 (16.5)	75,200 (41.5)		
Part-time employed	2800 (9.2)	26,200 (14.5)		
Unemployed – looking for full time work	5500 (18.2)	16,900 (9.3)		
Unemployed – looking for part-time work	500 (1.7)	5500 (3.0)		
Not in labour force	16,500 (54.5)	57,300 (31.6)	10,752.8 (<.001)	.23

**Fig. 2:**
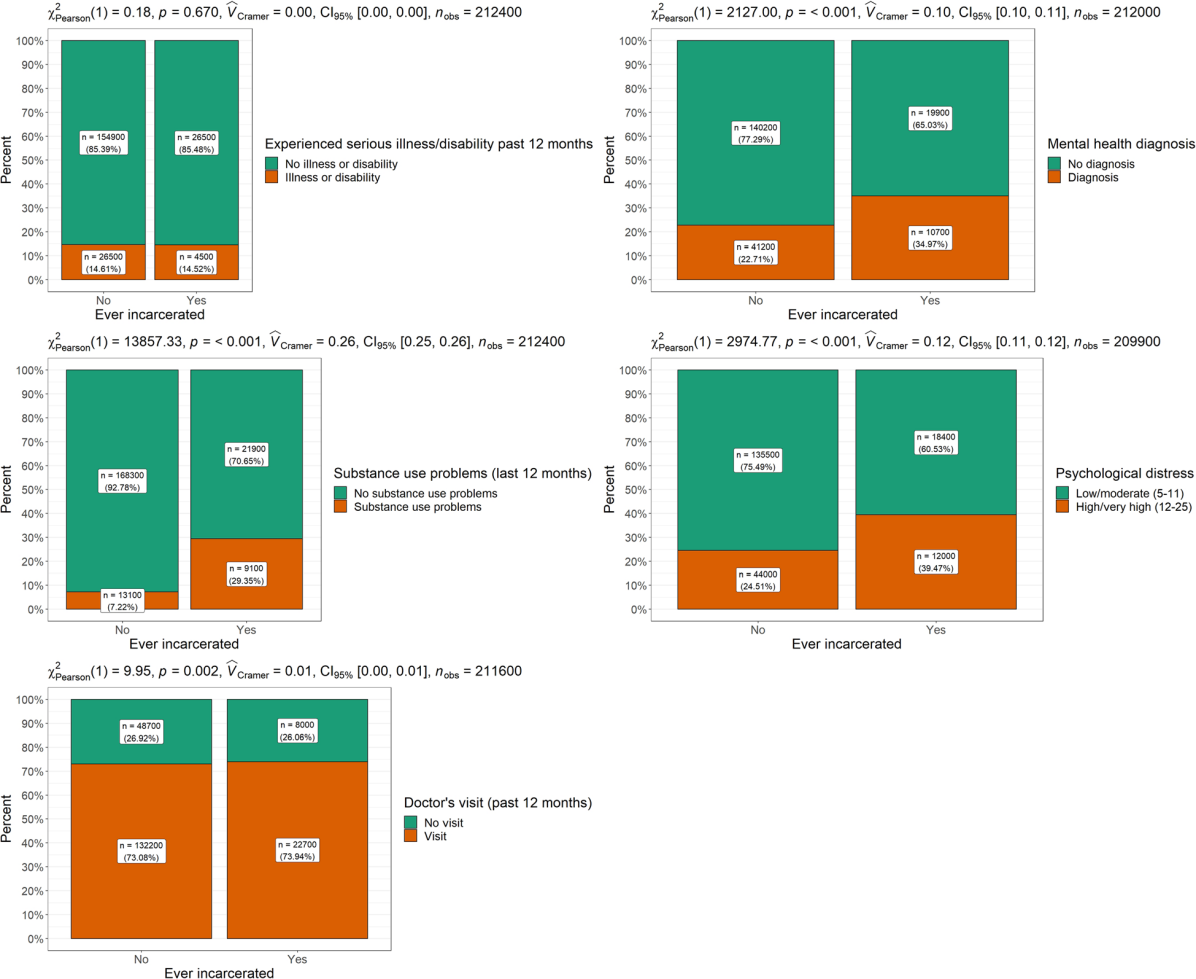
Health related variables by incarceration status

Health related variables are compared by incarceration status in Table 2 and Fig. 2. Reported experiences of serious illness or disability were similar across groups and were not statistically significant. All other tests were statistically significant. Ever-incarcerated individuals reported more mental health diagnoses and greater levels of high/very high psychological distress. Furthermore, ever-incarcerated individuals reported alcohol/drug problems at a rate over 400% higher than never-incarcerated individuals. The majority of both groups (73.9 and 73.1%, respectively) reported visiting a doctor over the last 12 months.

**Table 2:** Health related variables by incarceration status

	Ever- incarcerated *N* (%)	Never- incarcerated *N* (%)	*Χ*^2^ (*p* value)	*V*
Experienced serious illness/disability past 12 months				
No illness/disability	26,500 (85.5)^a^	154,900 (85.4)		
Illness/disability	4500 (14.5)^a^	26,500 (14.6)	0.2 (.67)	<.001
Mental health diagnosis				
No mental health diagnosis	19,900 (65.0)	140,200 (77.3)		
Mental health diagnosis	10,700 (35.0)^a^	41,200 (22.7)	2127.0 (<.001)	.10
Alcohol/drug problem last 12 months				
No substance use problems	21,900 (70.7)	168,300 (92.8)		
Substance use problems	9100 (29.4)	13,100 (7.2)	13,857.3 (<.001)	.26
Psychological distress				
Low/Moderate (5–11)	18,400 (60.5)	135,500 (75.5)		
High/very high (12–25)	12,000 (39.5)	44,000 (24.5)	2974.8 (<.001)	.12
Doctors visit last 12 months				
No visit to doctors	8000 (26.1)	48,700 (26.9)		
Visit to doctors	22,700 (73.9)	132,200 (73.1)	10.0 (.002)	.01

Note: ^a^ Numbers have a relative standard error of 25 to 50%

**Fig. 3:**
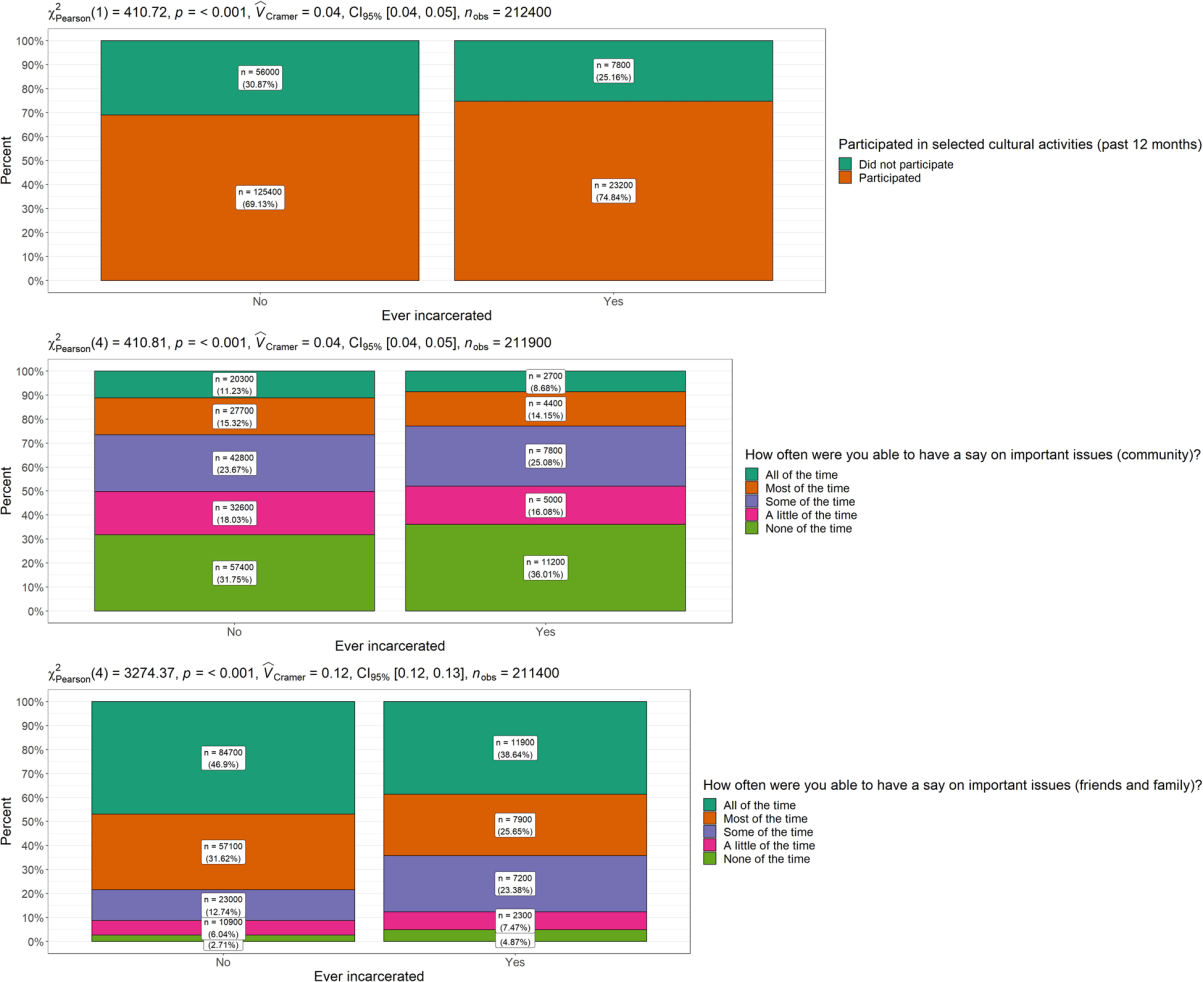
Social participation by incarceration status

Table 3 and Fig. 3 present variables relating to social participation by incarceration status. All comparisons produced statistically significant differences. Almost three-quarters of the ever-incarcerated group had participated in cultural activities over the last 12 months compared to almost 70% of the never-incarcerated group. Small percentage differences were identified by incarceration status for the variable relating to how often individuals had a say within community on important issues. The never-incarcerated group reported more frequent levels of contribution compared to the ever-incarcerated group. At the family/friends level, the never-incarcerated group more frequently ‘had a say’ on important issues.

**Table 3:** Social participation by incarceration status

	Ever-incarcerated *N* (%)	Never- incarcerated *N* (%)	*Χ*^2^ (*p* value)	*V*
Participated selected cultural activities last 12 months				
Did not participate	7800 (25.2)	56,000 (30.9)		
Participated	23,200 (74.8)	125,400 (69.1)	410.7 (<.001)	.04
How often able to have a say within community on important issues				
All of the time	2700 (8.7)	20,300 (11.2)		
Most of the time	4400 (14.2)	27,700 (15.3)		
Some of the time	7800 (25.1)	42,800 (23.7)		
A little of the time	5000 (16.1)	32,600 (18.0)		
None of the time	11,200 (36.0)	57,400 (31.8)	410.8 (<.001)	.04
How often able to have a say with family and friends on important issues				
All of the time	11,900 (38.6)	84,700 (46.9)		
Most of the time	7900 (25.7)	57,100 (31.6)		
Some of the time	7200 (23.4)	23,000 (12.7)		
A little of the time	2300 (7.5)^a^	10,900 (6.0)		
None of the time	1500 (4.9)	4900 (2.7)	3274.4 (<.001)	.12

Note: ^a^ Numbers have a relative standard error of 25 to 50%

**Fig. 4:**
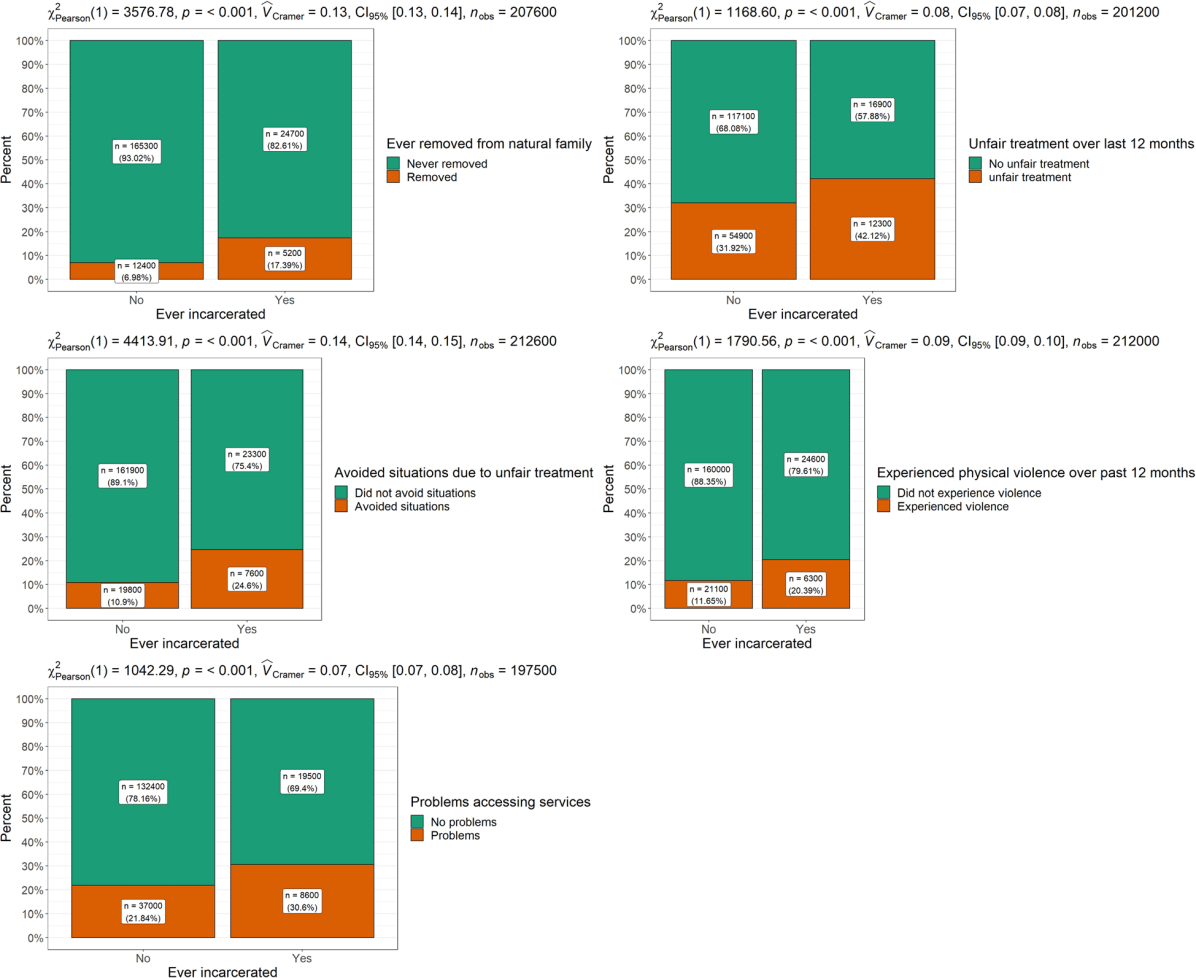
Social challenges by incarceration status

Table 4 and Fig. 4 present variables concerning social challenges. Differences by incarceration status were significant across all of the variables examined. A large discrepancy was identified for the variable ‘ever removed from natural family’. More than 17% of the incarcerated group reported having been removed from their natural family compared with 7% of those who had never been incarcerated. A similar discrepancy was observed for the variable relating to unfair treatment because of Indigenous status. A higher proportion of the ever-incarcerated group reported unfair treatment over the last 12 months (42%) compared to the never-incarcerated group (32%). A similar trend was observed for the variable ‘whether avoided situations due to unfair treatment because of Indigenous status’ (25% ever-incarcerated vs. 11% never-incarcerated). Regarding physical violence, over 20% of the ever-incarcerated group reported an occurrence over the last 12 months compared to 11.7% of the never-incarcerated group. More members of the ever-incarcerated group also reported having problems accessing services (31% ever-incarcerated vs. 22% never-incarcerated).

**Table 4:** Social challenges by incarceration status

	Ever-incarcerated *N* (%)	Never- incarcerated *N* (%)	*Χ*^2^ (*p* value)	*V*
Ever removed from natural family				
Never removed	24,700 (82.6)	165,300 (93.0)		
Ever removed	5200 (17.4)	12,400 (7.0)	7272.9 (<.001)	.13
Unfair treatment over last 12 months				
No unfair treatment	16,900 (57.9)	117,100 (68.1)		
Unfair treatment	12,300 (42.1)	54,900 (31.9)	2102.5 (<.001)	.07
Avoided situations due to unfair treatment				
Did not avoid situations	23,300 (75.4)	161,900 (89.1)		
Avoided situations	7600 (24.6)	19,800 (10.9)	8856.2 (<.001)	.15
Experienced physical violence over past 12 months				
Did not experience physical violence	24,600 (79.6)	160,000 (88.4)		
Experienced physical violence	6300 (20.4)	21,100 (11.7)	3570.5 (<.001)	.09
Problems accessing services				
Did not have problems accessing services	19,500 (69.4)	132,400 (78.2)		
Had problems accessing services	8600 (30.6)	37,000 (21.8)	2046.5 (<.001)	.07

**Fig. 5:**
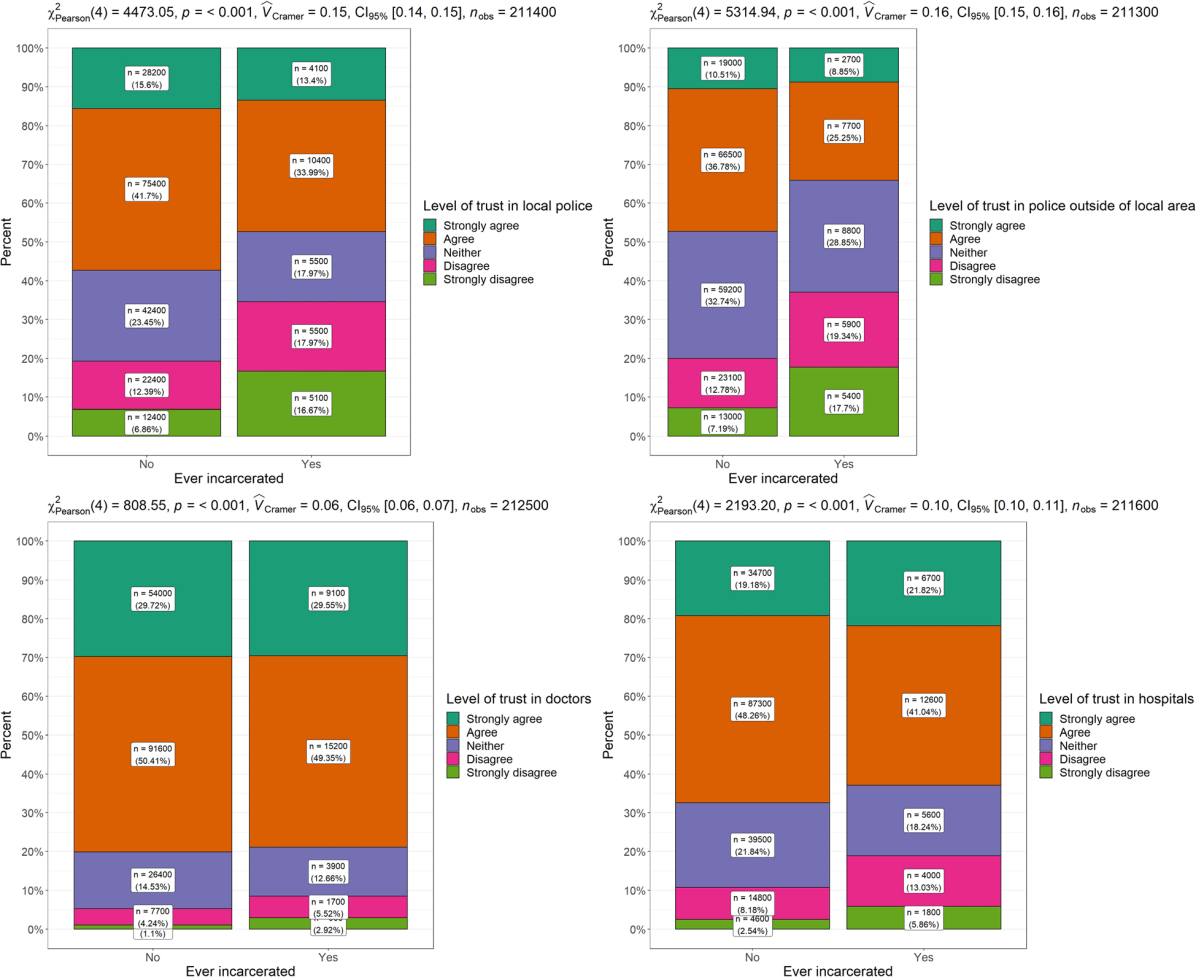
Trust in health and law enforcement institutions by incarceration status

Table 5 and Fig. 5 present variables relating to trust in health and law enforcement institutions. All comparisons examined were significant. Regarding trust in police, a greater percentage of never-incarcerated individuals expressed greater levels of trust compared to ever-incarcerated individuals. Levels of trust in own doctor were comparable by incarceration status with minor differences at the ‘strongly disagree’ level. However, larger differences were identified regarding trust in hospitals. Here, a larger percentage of never-incarcerated individuals agreed with the proposition compared to ever-incarcerated individuals.

**Table 5:** Trust in institutions by incarceration status

	Ever-incarcerated *N* (%)	Never- incarcerated *N* (%)	Χ^2^ (*p* value)	*V*
Level of trust in police in local area				
Strongly agree	4100 (13.4)^a^	28,200 (15.6)		
Agree	10,400 (34.0)	75,400 (41.7)		
Neither	5500 (18.0)	42,400 (23.5)		
Disagree	5500 (18.0)	22,400 (12.4)		
Strongly disagree	5100 (16.7)	12,400 (6.9)	4473.1 (<.001)	.15
Level of trust in police outside of local area				
Strongly agree	2700 (8.9)^a^	19,000 (10.5)		
Agree	7700 (25.3)	66,500 (36.8)		
Neither	8800 (28.9)	59,200 (32.7)		
Disagree	5900 (19.3)	23,100 (12.8)		
Strongly disagree	5400 (17.7)	13,000 (7.2)	5314.9 (<.001)	.16
Level of trust in own doctor				
Strongly agree	9100 (29.6)	54,000 (29.7)		
Agree	15,200 (49.4)	91,600 (50.4)		
Neither	3900 (12.7)	26,400 (14.5)		
Disagree	1700 (5.5)	7700 (4.2)		
Strongly disagree	900 (2.9)^a^	2000 (1.1)	808.6 (<.001)	.06
Level of trust in hospitals				
Strongly agree	6700 (21.8)	34,700 (19.2)		
Agree	12,600 (41.0)	87,300 (48.3)		
Neither	5600 (18.2)	39,500 (21.8)		
Disagree	4000 (13.0)	14,800 (8.2)		
Strongly disagree	1800 (5.9)^a^	4600 (2.5)	2193.2 (<.001)	.10

Note: ^a^ Numbers have a relative standard error of 25 to 50%

## Discussion

This study aimed to ascertain the existence and acuteness of an ‘incarceration gap’ within the Indigenous Australian population. A number of socio-economic, environmental and clinical factors were compared by life-time incarceration status. As expected, disparities were observed across numerous domains, the most pronounced being for educational obtainment, labour force participation and drug/alcohol problems. These three factors have demonstrated associations with justice-involvement for Indigenous populations in prior research [13–15, 28, 34]. They are also universally common risk factors for offending [43] and often characterise the risk profiles of non-Indigenous prisoners.

Almost half of the ever-incarcerated group in this study had not completed year 10. This rate was lower than the non-Indigenous male Australian population completion rate of 87.9% [44], but it was also substantially lower than the year 10 completion rate of never-incarcerated Indigenous Australians. Similar findings were obtained for labour force participation. While the never-incarcerated population’s work force status (56%) was trending towards that of the general Australian male population (65%) [45], only one-quarter of the ever-incarcerated population reported being employed. In addition, more than half of ever-incarcerated individuals reported that they were not in the labour force (for e.g., retired, voluntarily inactive, home duties, disabled, permanently unable to work). The link between lower educational obtainment and unemployment is well documented [46]. Barriers to ongoing employment are often exacerbated for those with a criminal record. Halving the Indigenous/non-Indigenous school attainment and employment gaps are key CTG targets. However, these aspirations - which have demonstrated some progress – must also contend with the substantial disparities observed here within the Indigenous population by incarceration status.

The third major discrepancy by incarceration status was possessing substance use problems over the past 12 months. Almost 30% of ever-incarcerated individuals reported such problems compared to just 7.2% of never-incarcerated individuals. Alcohol and drug abuse are major contributors to Indigenous offending and other problem behaviours [47, 48]. For example, a greater proportion of Indigenous prisoners report being affected by substances at the time of their offending compared to non-Indigenous prisoners [19]. In summary, the incarceration gap appears to be widest for the risk factor triad of under-education, unemployment and substance abuse.

A number of other factors distinguished ever-incarcerated individuals from never-incarcerated individuals, though to a lesser extent. Perhaps unsurprisingly, greater proportions of ever-incarcerated individuals distrusted police. It is not uncommon for individuals with repeat justice involvement to have negative attitudes towards law enforcement and the criminal justice system at large, often due to negative experiences and interactions with police. Attitudes towards police were more favourable for never-incarcerated individuals with less than 20% expressing distrust. The latter finding is more aligned with attitudes towards police for the general Australian population [49]. Another difference by incarceration status was the avoidance of situations due to perceived unfair treatment which was more common among the ever-incarcerated. Although there was some difference in experiences of unfair treatment (42% vs. 32%), a broader concern was that large minorities of both ever and never-incarcerated groups reported receiving unfair treatment. These proportions are similar to those found in other international representative surveys of Indigenous peoples [50]; and point to ongoing experiences of racism in Australian society. Given their higher levels of education and labour force participation, never-incarcerated individuals may possess an enhanced capacity to navigate perceived discriminatory experiences. It is also possible that perceived experiences of unfair treatment may be augmented for those who inhabit multiple disadvantaged or stigmatised sub-groups (i.e., Indigenous x justice-involved; justice-involved x low socio-economic status x Indigenous).

Ever-incarcerated individuals were also more dispersed across urban and remote regions. This contrasted with never-incarcerated individuals who were more concentrated in major urban areas. Reports have detailed that people living in remote and rural regions are more likely to be exposed to violence [51] and engage in the harmful use of substances [52]. Indigenous Australians are more likely to live in rural and remote areas of the country compared to non-Indigenous Australians [42]. Moreover, study results indicated that almost three-quarters of ever-incarcerated individuals live in Indigenous-only households compared to 50% of never-incarcerated individuals. This finding may be a reflection of rurality and/or socio-economic status. Evidence suggests that mixed Indigenous/non-Indigenous couples are more likely to reside in major urban areas and tend to be economically better-off [4, 53, 54]. Another notable difference by incarcerated status was mental health factors. Higher proportions of the ever-incarcerated group reported high to very high levels of distress and a life-time mental health diagnosis (35%). Prior research has pointed to the elevated distress levels of Indigenous prisoners [34, 37, 55], and Australian prisoners in general [56, 57]. The proportion of never-incarcerated individuals with a mental illness (22.7%) is in line with Australian male general population estimates (approx. 18%) [58]. Furthermore, findings imply that proportionally, ever-incarcerated individuals have endured more adverse life experiences. An alarming 17.4% reported being removed from their natural family. It is unknown as to the nature or circumstances of these removals. Historically, many Indigenous children were removed from their families through a systematic government policy known as the ‘Stolen Generation’ [59]. In recent decades, reports have detailed the disproportionate rates of Indigenous young people who have been removed from their parents or primary care-givers ostensibly due to a substantiated risk of harm [60]. Rates of child removal are also reportedly higher in rural and remote areas [61] where ever-incarcerated individuals are more likely to reside. Additionally, 1 in 5 ever-incarcerated individuals reported experiencing physical violence over the past 12 month compared to approximately 1 in 10 never-incarcerated individuals. However, the never-incarcerated rate is still markedly higher than the Australian general population rate [62]. Indigenous people experience violence at twice the rate of non-Indigenous people [63]. High-risk alcohol use, justice-involvement, unemployment and being removed as a child have demonstrated strong associations with violent victimisation [64, 65].

Though reaching significance, several factors yielded minor effects. Reported instances of serious illness/disability over the past 12 months were comparable by incarceration status. These findings are perhaps understated given their restriction to instances within the past year. According to official estimates, almost a quarter of Indigenous Australians report living with a disability, which is higher than for non-Indigenous Australians [66]. Access to health services was unexpectedly similar across groups. Three-quarters of both incarcerated and non-incarcerated respondents reported visiting a doctor over the past 12 months. However, the frequency and nature of the visit was unknown. Nonetheless, this finding is within range of the male Australian general population (80%) [67] and the majority of respondents across incarceration status appeared to trust their own doctor. Both groups were also more inclined to trust hospitals, though ever-incarcerated individuals reported marginally higher levels of distrust. A small effect was observed for the variable ‘problems accessing services’ – almost one-third of the incarcerated group reporting this concern compared to just over 20% of the never-incarcerated group. Health service accessibility problems have been linked to local availability, language barriers and long waiting times for Indigenous Australians, issues that are compounded in remote localities [68]. Ex-prisoners generally tend to visit doctors more than the general population due to more complex health needs [69], which underscores the concern that a notable minority of the ever-incarcerated group report difficulties accessing services.

The majority of respondent’s reported participating in cultural activities over the past year with little difference found by incarceration status. Some evidence has pointed to the protective influence of cultural involvement/connection for Indigenous people in custody [35, 37, 38]. However the temporal relationship between cultural participation and imprisonment was unknown for ever-incarcerated individuals. A meaningful relationship between experiences of racism and justice-involvement in Australia has not been established. Finally, family and community agency differed somewhat by incarcerated status. An insignificant distinction was identified at the community level – the majority of both groups reported having little to no say on important community issues. Much has been written on the lack of community consultation when devising policies directed at improving the lives of Indigenous Australians. However, rates of Indigenous participation (i.e., ‘having a say’) in community issues are similar to those reported by the general Australian population [70]. In contrast, a larger effect was identified at the family level. Ever-incarcerated individuals ‘had a say with family and friends on important issues’ with less regularity compared to never-incarcerated individuals. This may be a function of unavailability due to justice-involvement or perhaps a consequence of family estrangement from the offending relative.

The study had a number of limitations. The Australian Bureau of Statistics (ABS) TableBuilder platform permits users to extract cross-tabulations of simple aggregate information. As such, we were not able to conduct multivariate analyses that would allow for the examination of differences between the incarcerated and non-incarcerated group on one variable whilst adjusting for differences on other variables. However, this was not the intention of this analysis, which was to ascertain collective gaps within the Indigenous population. As such, ABS Table Builder was a useful device for cross-tabulating the aggregate data. In order to examine the correlates of imprisonment using multivariate analyses – an exercise we recommend for this sample - de-identified microdata is required. Our analysis did not include females. We believe this warrants specialised exploration as there may be unique differences within the Indigenous female population by incarceration status as compared to males. Moreover, Indigenous women are one of the fastest growing cohorts in Australian prisons [7]. Particular variables (i.e., Doctor visits past 12 months) provide somewhat limited information without a further qualitative understanding of the experiential nature of the episode. For example, it is possible that one group was more likely to experience suboptimal interactions with medical staff compared to the other group. The year 10 completion rate in the study may be marginally understated given that a small number of 15 and 16 year olds may have still been completing year 10 at the time of the survey. Access to time sensitive variables (pre and post incarceration) and more detailed justice system information (number of times in prison, time spent in prison) would be useful to unpack the relationship between justice-involvement and poorer social and health outcomes. We also recommend statistical comparisons with the non-Indigenous population where possible.

## Conclusions

There appears to be evidence for an ‘incarceration gap’ within the Indigenous Australian population across a number of important health and socio-economic markers. As observed, the never-incarcerated group – which comprises approximately 80% of the overall Indigenous male population - yielded aggregate proportions for numerous variables that approximated or matched general Australian population estimates. The gap would arguably be more pronounced if the sample included Indigenous Australians who were in custody at the time of the survey. Justice targets have been proffered in response, to control the ‘compounding’ effects incarceration has on individual and community disadvantage. Many of the salient correlates of Indigenous justice-involvement could perhaps be addressed through existing CTG targets (i.e, early childhood development, school completion and employment initiates). Other key factors such as substance abuse and early exposure to trauma/maltreatment will also require attention in order to realise justice targets. It is plausible that justice-involvement itself, partly undergirds or exacerbates the above risk factors rather than the reverse. Prior research indicates that both prior imprisonment *and* certain risk factors ‘prior-to-prison’ (i.e., removed as a child, low educational attainment) predict re-imprisonment for Indigenous Australians [71]. Longitudinal analyses which collect information (i.e., behavioural, environmental, biological) from early childhood with follow-up to adulthood (using criminal records) are recommended to delineate these temporal effects.

## Data Availability

The aggregate data extracted and analysed during the current study is publicly available from the ABS website (www.abs.gov.au).

## Abbreviations

NATSISS: National Aboriginal and Torres Strait Islander Social Survey
CTG: Close the Gap
ABS: Australian Bureau of Statistics
